# Using Partial Directed Coherence to Study Alpha-Band Effective Brain Networks during a Visuospatial Attention Task

**DOI:** 10.1155/2019/1410425

**Published:** 2019-09-03

**Authors:** Zongya Zhao, Chang Wang

**Affiliations:** ^1^School of Biomedical Engineering, Xinxiang Medical University, Xinxiang 453003, China; ^2^Key Lab of Neurosense and Control, Xinxiang Medical University, Xinxiang 453003, China

## Abstract

Previous studies have shown that the neural mechanisms underlying visual spatial attention rely on top-down control information from the frontal and parietal cortexes, which ultimately amplifies sensory processing of stimulus occurred at the attended location relative to those at unattended location. However, the modulations of effective brain networks in response to stimulus at attended and unattended location are not yet clear. In present study, we collected event-related potentials (ERPs) from 15 subjects during a visual spatial attention task, and a partial directed coherence (PDC) method was used to construct alpha-band effective brain networks of two conditions (targets at attended and nontargets at unattended location). Flow gain mapping, effective connectivity pattern, and graph measures including clustering coefficient (*C*), characteristic path length (*L*), global efficiency (*E*_global_), and local efficiency (*E*_local_) were compared between two conditions. Flow gain mapping showed that the frontal region seemed to serve as the main source of information transmission in response to targets at attended location while the parietal region served as the main source in nontarget condition. Effective connectivity pattern indicated that in response to targets, there existed obvious top-down connections from the frontal, temporal, and parietal cortexes to the visual cortex compared with in response to nontargets. Graph theory analysis was used to quantify the topographical properties of the brain networks, and results revealed that in response to targets, the brain networks were characterized by significantly smaller characteristic path length and larger global efficiency than in response to nontargets. Our findings suggested that smaller characteristic path length and larger global efficiency could facilitate global integration of information and provide a substrate for more efficient perceptual processing of targets at attended location compared with processing of nontargets at ignored location, which revealed the neural mechanisms underlying visual spatial attention from the perspective of effective brain networks and graph theory for the first time and opened new vistas to interpret a cognitive process.

## 1. Introduction

We can voluntarily limit our visual attention to a specific location in the visual field without changing the direction of eye gaze, and this visual spatial attention can improve perceptual processing of stimulus at attended location compared with processing of stimulus at ignored location [1–3]. Although visual spatial attention has been studied by different technologies and methodologies for many years, its neural mechanisms are still not well understood. Studies utilizing functional magnetic resonance imaging (fMRI) have consistently shown that two attention networks including the dorsal attention network (DAN) and the ventral attention network (VAN) are involved in visuospatial attention [1, 4, 5]. The DAN is mainly composed of intraparietal sulcus (IPS), superior parietal lobule, and frontal eye field (FEF) and shows increased blood-oxygenation-level-dependent (BOLD) signal when subjects voluntarily deploy their visual attention towards a target [4, 6–8]. The VAN, mainly consisting of the temporoparietal junction (TPJ) and the ventral frontal cortex, is thought to facilitate stimulus detection, particularly when unexpected stimuli are present [4–7, 9]. However, these two attention networks are not absolutely independent, and normal attentional function requires collaboration between the DAN and the VAN [10, 11].

At present, it is widely believed that visuospatial attentional processing relies on top-down control information from selective attentional control regions including the frontal and parietal cortexes to the visual cortex [7, 12–20]. The initial evidence comes from clinical observation where patients with parietal cortex lesions lost the ability to direct attention to one side of visual space [21], and subsequent studies from magnetoencephalographic (MEG) imaging [14], fMRI [22], and EEG [23] confirmed the top-down control effects from regions including FEF and IPS to the visual occipital cortex during visual spatial attention tasks. Due to the advantages of high temporal resolution, event-related potential (ERP) technique was used to investigate the transient changes of cognition process during visual spatial attention tasks, and results demonstrated that stimuli occurred at the attended location can elicit larger ERP components such as P1/N1 compared with stimuli at ignored location [24–26], showing that top-down control effects could ultimately amplify the sensory processing of stimulus occurred at the attended location relative to those at unattended location. Although there have existed a lot of studies on neural mechanisms of visual spatial attention, the changes in the brain networks during a visual spatial attention task are still far from being understood.

Based on the above findings, we propose that the process of visual attentional selection involves a distributed network including the occipital, parietal, temporal, and frontal cortexes and is a result of global integration of information among different brain areas. Therefore, it is necessary to study the effective brain networks and their topologic properties in response to targets at attended location and nontargets at unattended location. At present, many neuroimaging techniques such as fMRI, MEG, and EEG can be used to study brain networks [19, 27]. EEG still remains the most widespread technique so far due to its high time resolution and cheapness. The traditional methods for constructing brain networks such as correlation, synchronization, and coherence can only detect directionless functional connectivity among different brain regions. In the past few decades, several complicated EEG analysis methods have been proposed to measure the directional flows of information or effective connectivity among different brain areas [28–32]. Partial directed coherence (PDC) [29], one of the most commonly used methods to construct effective connectivity, is a full multivariate spectral measure used to determine the directed influences of Granger causality [33] between any given signals in a multivariate set, and it has been successfully applied in measuring the multichannel directed cortical interactions [27, 32, 34–36]. In addition, through the use of graph theory analysis, the topological characteristics of brain networks can be well revealed [37–40].

The aim of the present study was to investigate the brain networks and their topologic properties during visual spatial attention and further reveal the neural mechanisms underlying visual spatial attention from the perspective of effective brain networks and graph theory. In particular, we would like to reveal two main questions from the study: (1) What is the difference in effective brain connectivity pattern between target and nontarget condition? (2) What is the difference in topologic properties of brain networks between two conditions?

We hypothesized that the brain exhibited higher global efficiency of information integration across different brain areas in response to targets at attended location than in response to nontargets, which ultimately leads to improved perceptual processing of stimulus at attended location compared with processing of stimulus at ignored location. In order to test this hypothesis and answer above questions, we collected ERP data from 15 healthy subjects during a visual spatial attention task. Then, a partial directed coherence (PDC) method was used to construct alpha-band effective brain networks of two conditions (targets at attended and nontargets at unattended location). Based on the constructed effective networks, flow gain mappings were proposed to assess the role of the specific brain region involved in the visuospatial attention processing, and effective connectivity pattern was used to show the information flows among different brain regions. Finally, the topological parameters of the constructed brain networks were characterized with graph measures, and we mainly studied the difference in topologic properties of the effective brain networks between two conditions. To the best of our knowledge, this is the first study that revealed the neural mechanisms underlying visual spatial attention from the perspective of effective brain networks and graph theory.

## 2. Methods and Materials

### 2.1. Subjects and Experiment Design

**Figure 1:**
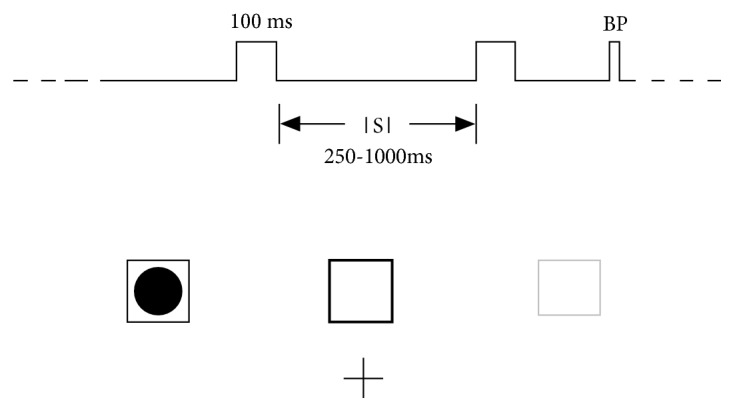
Schematic view of the experiment design (BP: button press; ISI: interstimulus interval; filled circle stands for stimuli; lightly shaded box stands for the attended location).

15 young subjects (8 males, 7 females; mean age: 21.2 ± 1.6; all right handed) with normal vision participated in the experiment, and an informed consent form was signed by all participants before the experiment. The experiment is a classical visual spatial attention task that is modified according to reference [41] (Figure 1): EEG data were collected from subjects who attended to randomized sequences of filled round disks appearing briefly inside one of the three empty squares that were constantly displayed 1.0 cm above a central fixation cross. The three empty 2.0 cm squares were constantly displayed on a black background at horizontal visual angles of 0°, +4°, and -4°from fixation cross, respectively. During each block of trials, one of the three empty squares was colored red and the other two were colored green. The red square indicated the location to be attended. The location was counterbalanced across blocks. One filled round disk was displayed for 120 ms within one of the three squares in a pseudorandom sequence with interstimulus intervals (ISI) of 250-1000 ms in four equiprobable 250 ms steps. Subjects were asked to maintain visual fixation on the central cross and respond only to stimuli (filled round disks) occurred in the attended square (the red square). Subjects pressed a button as soon as possible once the stimuli occurred in the attended square. For each block of trials, a total of 15 target and 60 nontarget trials were collected, and there were a total of 20 blocks for each subject. Subjects were given 1 min breaks between blocks.

### 2.2. EEG Recording and Preprocessing

The EEG data were recorded at a sampling rate of 1000 Hz from 16 Ag/Agcl electrodes (F3, Fz, F4, T7, C3, Cz, C4, T8, P7, P3, Pz, P4, P8, O1, Oz, and O2) that were mounted on the scalp with a 32-channel EEG cap (NeuroScan QuikCap) according to the 10-20 standard system. All channels were referenced to the right mastoid with input impedance below 5 K*Ω*.

Offline EEG preprocessing was carried out by using MATLAB 7.7.0 R2010a software (MathWorks Inc., USA) equipped with the EEGLAB toolbox [42]. Firstly, a 0.5-40 Hz zero-phase band-pass filter was applied. Then, ocular and prominent muscle artifacts were removed by means of independent component analysis (ICA). Specifically, the ICA was applied to the continuous EEG signals of each subject before extracting epochs of target and nontarget and was conducted by using the “binica” algorithm embedded in the EEGLAB toolbox with default parameters. We visually checked each component's scalp map and power spectrum to determine whether it was an artifact component, and the average number of artifact components was 1.8 ± 0.6 (mean ± std) for 15 subjects. Subsequently, the continuous EEG signal was downsampled to 250 Hz and divided into epochs time locked to the stimuli (−1.0 s to 1.2 s) which were baseline (−0.1 s to 0 s) corrected. Then, an extreme value of ±80 *μ*V was applied to further remove epochs which may contain artifacts. In addition, for target trials, a response time longer than 700 ms and shorter than 100 ms was considered as a lapse and coincidence, respectively, and the corresponding epochs were removed. Nontarget trials followed by a response were also discarded. Finally, we collected an average of 206 artifact-free target epochs and an average of 1048 artifact-free nontarget epochs for each subject. In order to exclude any bias from the unequal number of trials in both experimental conditions, we randomly selected 190 trials from the collected artifact-free target trials and artifact-free nontarget trials, respectively. Finally, 190 trials per subject/condition were remained for further analysis.

### 2.3. Event-Related Spectral Perturbation (ERSP) Calculation

ERSP is defined as the logarithm of the ratio of spectral power at a specific time point to that of the average spectral power over a reference period. Here, we estimated the spectral power change of whole epoch (−1.0 s to 1.2 s) relative to the baseline (−0.1 s to 0 s). ERSP of a single trial was carried out with the Morlet wavelet (EEGlab *newtimef* function), spanning 40 linearly spaced frequencies from 3.9 Hz to 30 Hz (from 3 cycles at 3.9 Hz to 11.4 cycles at 30 Hz) over a time course of 200 linearly spaced time points. Finally, for every epoch or trial, we obtained ERSP with a 2D matrix of 40 (frequency points) × 200 (time points).

### 2.4. Effective Connectivity Construction

Partial directed coherence (PDC) is a full multivariate spectral measure used to determine the directed influences of Granger causality between any given signals in a multivariate set [29, 43]. Let *X*(*n*) = [*x*_1_(*n*), *x*_2_(*n*), *x*_3_(*n*), ⋯,*x*_*N*_(*n*)]^*T*^ represent an N-channel EEG signal (*N* = 16 in this study), then a MVAR model with order *p* for *X*(*n*) could be expressed as

(1) $$\begin{array}{c} X\left(n\right)=\overset{p}{\underset{r=1}{\sum }}A_{r}X\left(n-r\right)+W\left(n\right), \end{array}$$

where  *W*(*n*) is a multivariate uncorrelated noise vector, *A*_*r*_ is the coefficient matrix, and *p* is the order of the MVAR model, which can be determined by using the Akaike information criterion (AIC)

(2) $$\begin{array}{c} \text{AIC}\left(p\right)=2\log\left[\det\left(\Sigma \right)\right]+\frac{2N^{2}p}{N_{\text{total}}}, \end{array}$$

where det(*Σ*) denotes the covariance matrix of the noise vector *W*(*n*) and *N*_total_ is the total number of EEG samples in all trials. In the present study, *p* ranges from 10 to 15 for all subjects. Specifically, the model order (mean/std) was 11 ± 2.2 and 13 ± 3.1 for nontarget condition and target condition, respectively. To estimate *A*_*r*_, equation (1) can be multiplied by *X*^*T*^(*n* − *k*), where *k* = 1, 2, ⋯, *p*, to obtain the Yule-Walker equations

(3) $$\begin{array}{c} \overset{p}{\underset{r=1}{\sum }}A_{r}R\left(-k+r\right)=0, \end{array}$$

where *R*(*m*) = <*X*(*n*) *X*^*T*^(*n* + *m*)> are the covariance matrices of all *X*(*n*) of lag *m*. A solution for *A*_*r*_ can be obtained using the Levinson-Wiggins-Robinson (LWR) algorithm [44]. Once the MVAR model has been estimated, a representation of Granger causality in the frequency domain can be obtained from the difference between the N-dimensional identity matrix I and the Fourier transform of the coefficient series *A*_*r*_(*r* = 1, 2, ⋯, *p*)

(4) $$\begin{array}{c} A\left(f\right)=I-\overset{p}{\underset{r=1}{\sum }}A_{r}e^{-2jfr\pi } \end{array}$$

Then, the directional flow of information at frequency *f* from channel *j* to channel *i* can be defined as

(5) $$\begin{array}{c} \text{PDC}\left(i,j,f\right)=\frac{\left|A_{ij}\left(f\right)\right|}{\sqrt{\sum_{k}A_{kj}^{*}\left(f\right)A_{kj}\left(f\right)}}, \end{array}$$

where asterisk denotes matrix transposition and complex conjugation, and *A*_*ij*_(*f*)  are the elements of the matrix *A*(*f*).

In this study, we used epochs of [0 0.6 s] after stimulus onset to compute PDC values. For ERP data, many realizations of the same process are available; a modified adaptive procedure for estimating the MVAR model can be employed to increase the reliability of the model parameters [45]. Briefly, the steps to create an adaptive MVAR model of the *X* process are as follows:

1. Compute the covariance matrices *R*_*n*_(*m*)(*n* = 1, 2, ⋯, *T*) for *T* trials of the *X* process in the target and nontarget condition for each subject
2. Obtain the average covariance matrix from the *T* trials: $\bar{R}\left(m\right)=\sum_{n=1}^{T}R_{n}\left(m\right)/T$
3. Replace the *R*(*m*) in the Yule–Walker equation (3) with $\bar{R}\left(m\right)$ and calculate the *A*_*r*_
4. Calculate PDC using *A*_*r*_ and equation (5)

In order to test the significance of PDC values, a nonparametric statistical test using surrogate data was implemented in the present study [46]. Briefly, the original time series from each epoch were transformed to the Fourier space, in which the phases are randomly shuffled without changing the magnitude. The surrogate data in the Fourier space are then transformed back to the time domain. This process of phase shuffling preserves the spectral structure of the time series and is suited for PDC analysis which is a measure of frequency-specific causal interactions. The PDC values were recalculated using the obtained surrogate data. An empirical distribution of PDC values under the null hypothesis of no causal relationships was obtained by repeating the shuffling and PDC estimation procedures 1000 times. Based on this empirical distribution, the PDC values were considered to be a real connection when they were above the threshold (*p* = 0.05).

In addition, because lots of studies have confirmed the important role of alpha band (7-14 Hz) in visuospatial attention processing [19, 24, 47–51], so we restricted our analysis in the alpha band in this study, and the PDC values were averaged over the alpha frequency band.

### 2.5. Graph Theoretical Analysis

The alpha-band PDC matrixes were converted into a directed binary graph by applying a sparsity of *T*, and the graphs can be characterized in terms of some basic graph measures including clustering coefficient (*C*), characteristic path length (*L*), global efficiency (*E*_global_), and local efficiency (*E*_local_). The clustering coefficient is a measure of the “cluster together degree” of nodes and is considered as a metric of the network segregation whereas the characteristic path length is defined as the average shortest paths for all possible pairs of nodes and is an indicator of global integration of information transmission. The global efficiency is a measure of the speed and efficiency of information transfer over a whole network whereas the local efficiency can be considered as the average efficiency of the local subgraphs, and it tells us how efficient is the communication between the first neighbors of node *i* when *i* is removed. Detailed descriptions and calculation methods for *L*, *C*, *E*_global_, and *E*_local_ could be found in some previously published literatures [52–56]. The small-world network is characterized by a similar path length and higher clustering coefficient compared to a random network, that is, *γ* = *C*_real_/*C*_random_ > 1, *λ* = *L*_real_/*L*_random_ ≈ 1, where *C*_random_ and *L*_random_ denote the average *C* and *L* of an ensemble of 1000 surrogate random networks which were derived from the experimental network by using the Markov-chain algorithm [57]. And the small-world index could be defined as *σ* = *γ*/*λ*, which is greater than 1 for a small-world network.

The sparsity *T* can be viewed as the ratio of the number of real effective connections to the number of all possible connections in this network. In this study, a wide sparsity range of 0.1-0.7 with a step of 0.01 was used to explore the features of the effective networks at different connection densities. We found that there existed significant differences between graph measures of brain networks under two experiment conditions in a *T* range of 0.31-0.55. Therefore, a sparsity of 0.38 was adopted in the study to reveal the topological features of the effective brain networks under two different experiment conditions.

Because sparsity is a biased and arbitrary network filtering scheme, therefore, we also tried using a data-driven network filtering scheme based on Orthogonal Minimal Spanning Tree (OMST), which filters brain connectivity networks based on the optimization between the global efficiency of the network and the cost preserving its wiring [58, 59]. By applying the OMST method, graph theoretical measures including clustering coefficient (*C*), characteristic path length (*L*), global efficiency (*E*_global_), local efficiency (*E*_local_), and small-world index (*σ*) were recomputed. In addition, in the present study, all graph theoretical measures were computed by using the Brain Connectivity Toolbox (BCT) toolbox [54].

### 2.6. Statistical Analysis

All statistical analysis was carried out using SPSS version 21.0 software (SPSS Inc., Chicago, IL). The data were expressed as mean ± standard error of mean (SEM). The Shapiro-Wilk test was used to test for normality of distribution. The comparison of ERSP between two conditions (target *vs.* nontarget) involving multiple time points was carried out using the paired sample *t* test with false discovery rate (FDR) correction for multiple comparisons [60, 61]. In order to determine the between-condition differences in the graph measures, we conducted a repeated-measures multivariate analysis of variance (MANOVA) with the following dependent variables: clustering coefficient, *C*; characteristic path length, *L*; global efficiency, *E*_global_; local efficiency, *E*_local_; and small-world index, *σ*. *p* < 0.05 showed that there existed a significant difference.

## 3. Results

### 3.1. Behavioral Results and ERSP Analysis

**Figure 2:**
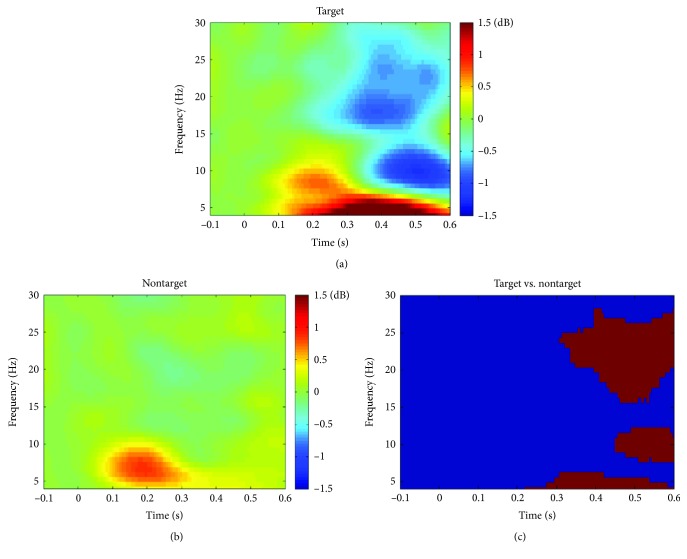
Spectral power changes averaged over 8 channels (P7, P3, Pz, P4, P8, O1, Oz, and O2) and plotted as 10log10 change over a baseline for target condition (a) and nontarget condition (b). (c) The comparison of ERSP between two conditions: red color indicates time-frequency regions significantly different between two conditions (*p* < 0.0021, FDR corrected) and blue color indicates no significant difference.

The mean reaction time of target condition was 389 ± 34 ms, and the mean detection rate was 95.7 ± 0.004%. Figures 2(a) and 2(b) showed the ERSP of target and nontarget condition, respectively.

Results showed that both targets and nontargets led to an increase of theta power over baseline and alpha/beta band desynchronization.  Figure 2(c) showed the comparison of ERSP between two conditions, which indicated that in response to targets, theta synchronization and alpha/beta desynchronization were stronger than in response to nontargets (*p* < 0.0021, FDR corrected).

### 3.2. Flow Gain Mapping and Effective Connectivity Pattern

The information flows among different brain regions can be computed based on the obtained alpha-band PDC matrixes. If *γ*_*ij*_ represent an element of PDC matrix, the inflow and outflow of channel *i* can be determined as ∑_*j*=1_^*N*^*γ*_*ij*_ and ∑_*j*=1_^*N*^*γ*_*ji*_, respectively, where *N* is the number of EEG channel. The inflow of channel *i* stands for the magnitude of all the incoming links from the other channels to channel *i* whereas the outflow indicates the magnitude of all the outgoing links from channel *i* to the others. We can define flow gain of channel *i* as the ratio of outflow to inflow. The flow gain of channel *i* can clearly show the role of channel *i* during information transmission process: a lower value of flow gain means that channel *i* serves as sink during information transmission to a greater extent whereas the channel is more apparent as a source as the value of flow gain increases.

**Figure 3:**
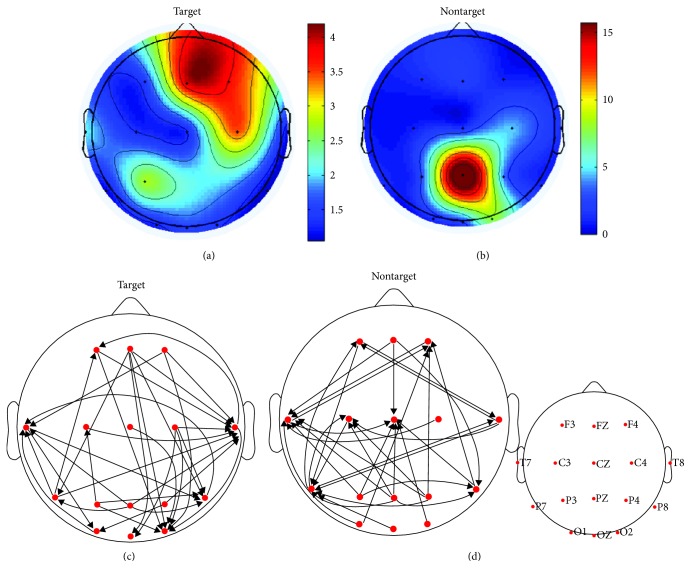
Flow gain mappings averaged over all subjects in response to target (a) and nontarget (b). Group average effective connection patterns of target (c) and nontarget (d) condition. The arrows stand for information flow direction. For better illustration purpose, only the top 15% of the maximum edges are shown.

Figures 3(a) and 3(b) showed the group average flow gain mapping in response to targets and nontargets, respectively. Results indicated that flow gain mapping of target condition was obviously different from that of nontarget condition. In particular, in response to targets, the most active regions that act as a hub and source of information communication are mainly located in the frontal cortex. However, the most active regions that serve as a source of information communication are mainly located in the parietal cortex for nontarget condition.

**Figure 4:**
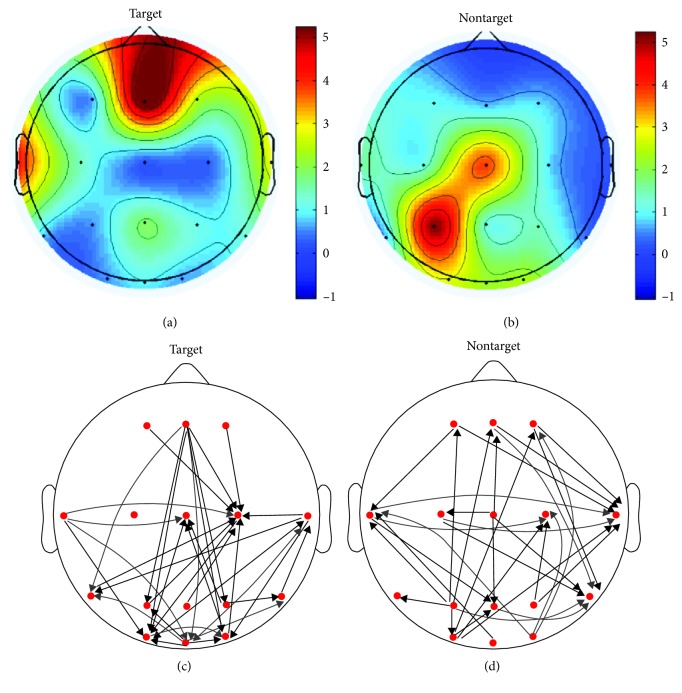
Flow gain mappings of one typical subject in response to target (a) and nontarget (b). One subject's effective connection patterns of target (c) and nontarget (d) condition. The arrows stand for information flow direction. For better illustration purpose, only the top 15% of the maximum edges are shown.

Figures 3(c) and 3(d) indicated the group average effective connectivity pattern in response to targets and nontargets, respectively, and the main difference between two conditions is as follows: (1) In response to targets, there existed obvious long-range connections from the frontal cortex to the visual cortex (occipital regions) whereas there almost no such long-range links for nontarget condition. (2) In addition to the frontal cortex, there also existed top-down links from the temporal and parietal cortexes to the visual cortex for target condition compared with nontarget condition. (3) For target condition, T8, T7, and O2 regions mainly serve as sinks of information communication whereas T7, P7, and Cz areas mainly act as sinks for nontarget condition.  Figure 4 showed the flow gain mappings and effective connection patterns of one typical subject in response to target (Figures 4(a) and 4(c)) and nontarget (Figures 4(b) and 4(d)). The above findings are mainly based on qualitative observations, and graph theoretical analysis was applied to quantitatively study the topological properties of the brain networks under two experiment conditions.

### 3.3. Topological Properties of the Brain Networks

In order to determine the between-condition differences in the graph measures, a repeated-measures MANOVA was carried out with the following dependent variables: clustering coefficient, *C*; characteristic path length, *L*; global efficiency, *E*_global_; local efficiency, *E*_local_; and small-world index, *σ*. The comparison results are summarized in Table 1. The main effect of condition (target vs. nontarget) showed a significant difference (*F*_5,24_ = 3.315, *p* = 0.020, partial *η*^2^ = 0.409). The mean *C* for target and nontarget condition was 0.419 ± 0.009 and 0.463 ± 0.012, respectively, and univariate tests revealed there existed a significant difference between two conditions (*F*_1,28_ = 8.400, *p* = 0.007, partial *η*^2^ = 0.231). In response to targets, the *L* (2.822 ± 0.108) of brain networks was significantly smaller than that (3.437 ± 0.128) of nontarget condition (*F*_1,28_ = 13.396, *p* = 0.001, partial *η*^2^ = 0.324). The *E*_global_ for target condition and nontarget condition was 0.562 ± 0.010 and 0.504 ± 0.012, respectively, and *E*_global_ for target condition was significantly longer than that of nontarget condition (*F*_1,28_ = 13.176, *p* = 0.001, partial *η*^2^ = 0.324). However, there was no significant difference between two conditions for *E*_local_ (*F*_1,28_ = 0.594, *p* = 0.447, partial *η*^2^ = 0.021) and *σ* (*F*_1,28_ = 1.156, *p* = 0.292, partial *η*^2^ = 0.040). In both conditions, the small-world index was larger than 1 (*σ* = 1.530 ± 0.134 for target condition, *σ* = 1.356 ± 0.091 for nontarget condition), which suggested that the brain networks in response to both targets and nontargets owned small-world properties.

The sparsity is a biased and arbitrary network filtering scheme that might add bias for condition comparisons and reduce the possibility of the reproducibility of the findings across studies from different research groups. Here, the graph theoretical measures were recomputed by applying a data-driven network filtering scheme based on OMST, and the results are summarized in Table 2. The main effect of condition (target vs. nontarget) showed a significant difference (*F*_5,24_ = 5.487, *p* = 0.002, partial *η*^2^ = 0.533). In response to targets, the *L* (2.689 ± 0.168) of brain networks was significantly smaller than that (3.510 ± 0.129) of nontarget condition (*F*_1,28_ = 15.602, *p* < 0.001, partial *η*^2^ = 0.358). The *E*_global_ for target condition and nontarget condition was 0.573 ± 0.012 and 0.497 ± 0.010, respectively, and *E*_global_ for target condition was significantly longer than that of nontarget condition (*F*_1,28_ = 23.998, *p* < 0.001, partial *η*^2^ = 0.462). However, there was no significant difference between two conditions for *C*, *E*_local_, and *σ*. We found that by using the OMST method, the graph measure *C* no longer shows a significant difference between target and nontarget condition.

## 4. Discussion

Here, we combined graph theoretical analysis and PDC method to study the brain effective connectivity and its topologic properties underlying visual spatial attention in healthy subjects for the first time. We observed that in response to targets, there existed obvious top-down connections from a distributed network of brain areas involved in attention control including the frontal, temporal, and parietal cortexes to the visual cortex compared with in response to nontargets. More importantly, we found that for target condition, the brain networks were characterized by significantly larger characteristic path length and global efficiency than nontarget condition, suggesting that larger characteristic path length and global efficiency could facilitate global integration of information and provide a substrate for more efficient perceptual processing of targets at attended location compared with processing of nontargets at ignored location. These results provided a new perspective for us to understand the neural mechanisms underlying visual spatial attention.

Oscillations in the brain play critical roles in visual spatial attention. Our ERSP results indicated that in response to targets theta synchronization and alpha/beta desynchronization were stronger than in response to nontargets (Figure 2). For alpha/beta (usually alpha and beta bands) band oscillation, lots of studies have proved that there existed an inverse relationship between alpha/beta power and behavioral selective attention through time and hypothesized that alpha/beta waves acted as an attention suppression mechanism in which brain regions processing irrelevant information utilize increased alpha/beta power [19, 24, 48, 62], which is consistent with our findings. In addition to alpha/beta band, comparison between two conditions indicated that theta synchronization was stronger during target processing, although processing of both nontargets and targets led to an increase of theta power over baseline, which was consistent with the results of other literatures [5, 62]. The above results showed that theta band and alpha/beta band might play different roles in modulating visual spatial attention.

It is widely believed that visuospatial attention relies on top-down control information from selective attentional control regions including the frontal and parietal cortexes to the visual cortex. Here, we tried to use flow gain mapping and effective brain connectivity pattern to qualitatively investigate the information communication among different brain areas during visual spatial attention. Our results showed that in response to targets, the most active regions that act as a hub and source of information communication are mainly located in the frontal cortex (Figure 3(a)), suggesting the important role of the frontal cortex in fast response to stimulus at attended location. Some researchers reported that prefrontal lesions reduced visually evoked EEG activity during a visual detection task for humans [63]. Subthreshold electrical stimulation of the frontal cortex while the animal performs an attention-demanding change detection task improved the animal's ability to detect small changes in luminance [64]. Moreover, Ruff et al. [65] found that in humans, transcranial magnetic stimulation of the frontal region altered BOLD responses in early visual areas, leading to enhanced responses to peripheral visual stimuli. In addition, lots of electrophysiological and neuroimaging studies have also confirmed the causal influences of frontal regions on visual areas in visual spatial attention [1, 16, 66, 67]. Here, we introduced the flow gain mapping for the first time to assess the role of the specific brain region involved in response to targets and found that the frontal cortex seemed to serve as the main sources of information transmission, which was consistent with the above-mentioned studies. However, in response to nontargets, the most active regions that serve as a source of information communication are mainly located in the parietal cortex (Figure 3(b)). Suzuki and Gottlieb [68] performed an experiment which compared prefrontal and parietal activity during a memory-guided saccade task with distractors and found that the neuron activity in the frontal cortex was strongly suppressed in response to distractors while the neuron activity in the parietal cortex was transiently greater in response to distractors than the sustained target response, which might explain our finding that the parietal cortex seemed to serve as the main sources of information transmission in response to nontargets. We inferred that the frontal and partial cortexes are specialized for different aspects of attention control, but further studies need to be carried out.

Although flow gain mapping was helpful for studying the overall information flow during a cognitive process, it did not show the detailed direction information. Here, effective connectivity pattern was applied. Our results indicated that in response to targets, there existed obvious connections from attention-related regions including the frontal, temporal, and parietal cortexes to the visual cortex (Figure 3(c)) whereas there almost no such links for nontarget condition (Figure 3(d)), showing that the top-down control was necessary for more efficient perceptual processing of targets at attended location compared with processing of nontargets at ignored location. Converging evidence has proved that the control of visual attention involved a distributed network encompassing the regions of occipital, parietal, temporal, and frontal cortexes [1, 7, 17, 66, 69, 70]. For example, studies from MEG imaging [14, 19] showed that in response to targets at attended location, various brain areas including the anterior cingulate cortex, left middle and inferior frontal gyri, left superior temporal gyrus, and inferior parietal lobule send top-down control information to early visual areas. In addition, study from fMRI [22] revealed that there were more top-down control information sending from FEF and IPS to the visual occipital cortex than bottom-up information from the visual cortex to FEF and IPS during a visual spatial attention task. In a word, our findings of effective connectivity pattern were consistent with these previous studies.

In recent years, graph theory analysis has been widely applied to study the topologic characteristics of brain networks. Previous studies have implied that the small-world network is considered to be one of the most appropriate models to balance local segregation and global integration in human brain [71]. The small-world network is characterized by a higher clustering coefficient compared to a random network and a shorter path length compared to a regular network, which allows for more efficient information transfer among distant brain regions. It was well-known that the characteristic path length *L* is defined as the average shortest paths for all possible pairs of nodes and stands for global efficiency of information integration across different brain areas. Our results showed that in response to targets, the *L* of brain networks was significantly smaller than that of nontarget condition, suggesting a more efficient information integration and communication across different brain regions for target condition compared with nontarget condition. A MEG study in healthy subjects showed that the cognitive effort drove normal brain networks to a less clustered configuration and more long-range synchronization [72]. Effective connectivity pattern analysis (Figure 2(c)) showed that in response to targets, there existed many long-range connections from the frontal cortex to the visual cortex whereas there almost no such long-range links for nontarget condition, which might explain the smaller *L* for target condition in our study. In addition, lots of previous studies have confirmed the important role of this long-range connections in visual spatial attention [1, 63, 65, 66]. The global efficiency *E*_global_ is also a measure of the speed and efficiency of information transfer over a whole network, and our results indicated that in response to targets, the *E*_global_ of brain networks was significantly larger than that of nontarget condition. In a word, we believed that smaller characteristic path length and larger global efficiency could facilitate global integration of information and provide a substrate for more efficient perceptual processing of targets at attended location compared with processing of nontargets at ignored location.

In addition, the small-world index of target condition was larger than that of nontarget condition, but there existed no significant difference. In both conditions, the small-world indexes were larger than 1, suggesting that the brain networks in response to both targets and nontargets owned small-world properties.

The present study has certain limitations. On the one hand, our study was based on low-density EEG recordings. Although some previous studies investigated topological properties of brain networks by using low-density EEG [40, 73], the node of the networks based on low-density EEG is relatively small. On the other hand, from a methodological point of view, our study converted effective connectivity based on PDC into binary graph, which would result in the loss of part of the information compared to weighted graph.

## 5. Conclusion

In summary, our results showed that the frontal region seemed to serve as the main source of information transmission in response to targets while the parietal region served as the main source in nontarget condition, and in response to targets, there existed obvious top-down connections from the frontal, temporal, and parietal cortexes to the visual cortex compared with in response to nontargets. More importantly, our results revealed that in response to targets, the brain networks were characterized by significantly smaller characteristic path length and larger global efficiency than in response to nontargets, which suggested a more efficient information integration and communication across different brain regions for target condition compared with nontarget condition. The present study combining effective connectivity and graph theory analysis provided helpful findings to reveal the neural mechanisms underlying visual spatial attention and also opened new vistas to interpret a cognitive process.

## Figures and Tables

**Table 1:** Comparisons of the graph measures between target and nontarget condition based on a sparsity method.

Graph measures	Target (mean ± SEM)	Nontarget (mean ± SEM)	Statistical analysis		
			*F*_1,28_	*p*	Partial *η*^2^
*C*	0.419 ± 0.009	0.463 ± 0.012	8.400	**0.007**	0.231
*L*	2.822 ± 0.108	3.437 ± 0.128	13.396	**0.001**	0.324
*E*_global_	0.562 ± 0.010	0.504 ± 0.012	13.176	**0.001**	0.320
*E*_local_	0.560 ± 0.009	0.574 ± 0.015	0.594	0.447	0.021
*σ*	1.530 ± 0.134	1.356 ± 0.091	1.156	0.292	0.040

Notes: values are expressed as mean ± SEM; *C* stands for clustering coefficient; *L* stands for characteristic path length; *E*_global_ represents global efficiency; *E*_local_ represents local efficiency; *σ* stands for small-world index; significant differences (*p* < 0.05) between two conditions are highlighted in bold.

**Table 2:** Comparisons of the graph measures between target and nontarget condition based on the OMST method.

Graph measures	Target (mean ± SEM)	Nontarget (mean ± SEM)	Statistical analysis		
			*F*_1,28_	*p*	Partial *η*^2^
*C*	0.475 ± 0.021	0.516 ± 0.022	2.034	0.165	0.068
*L*	2.689 ± 0.168	3.510 ± 0.129	15.602	**<0.001**	0.358
*E*_global_	0.573 ± 0.012	0.497 ± 0.010	23.998	**<0.001**	0.462
*E*_local_	0.550 ± 0.008	0.498 ± 0.011	0.329	0.571	0.012
*σ*	1.460 ± 0.092	1.346 ± 0.083	0.812	0.375	0.028

Notes: values are expressed as mean ± SEM; *C* stands for clustering coefficient; *L* stands for characteristic path length; *E*_global_ represents global efficiency; *E*_local_ represents local efficiency; *σ* stands for small-world index; significant differences (*p* < 0.05) between two conditions are highlighted in bold.

## Data Availability

The datasets analyzed during the current study are not publicly available due to the further analysis of the datasets being doing in our research but are available from the corresponding author on reasonable request.
